# The efficacy of combined music therapy and Tai Chi for major depressive disorder: Study protocol for a randomized controlled trial

**DOI:** 10.1097/MD.0000000000025241

**Published:** 2021-03-26

**Authors:** Huixian Xie, Yiping Tang, Fengmin Cheng, Jucai Chu

**Affiliations:** a Department of Nursing; b Department of Psychology, Taizhou Second People's Hospital, Zhejiang, China.

**Keywords:** major depression, music, protocol, randomized, Tai Chi

## Abstract

**Background:**

There are no clinical trials evaluating the efficacy of combined music and Tai Chi therapy for patients with major depression. Therefore, the primary objective of the present study was to evaluate the efficacy of Tai Chi and music therapy on psychological status and quality of life among patients with major depressive disorder in China.

**Methods:**

This work is a part of a comprehensive research project to assess and provide intervention that potentially improves psychological status and quality of life among patients with major depressive disorder. This research project has been received ethical approval from the Medical Research and Ethics Committee in Taizhou Second People's Hospital (no. tzey2020023). After the introductory briefing, the acceptance to take the presession questionnaire implied the participant's consent to participate in the study. Eligible participants are divided into 3 groups according to completely randomized design: combined group (music therapy + Tai Chi), music therapy alone group, and Tai Chi alone group. The analyses will be performed using SPSS 22.0.0 (SPSS Inc, Chicago, IL).

**Results:**

This protocol will provide a reliable theoretical basis for the following research.

**Conclusion:**

The sample came from a single health center. Therefore, the results cannot be generalized for the entire population.

**Trial registration:**

This study protocol was registered in Research Registry (researchregistry6597).

## 1 Introduction

Depressive symptoms affect about 35.1% of community-dwelling older persons and 65% of institutionalized older persons. In South-West China, depressive symptoms have been reported in 24.3% of community-dwelling older persons.^[1]^ Many individuals being treated for depression receive medication therapy only. Drug treatment, however, is often limited by lack of efficacy, side effects, and drug–disease interaction that are particularly problematic among frail older persons. It is, therefore, important to develop efficient, convenient, and cost-effective solutions of reducing the burden of depressive symptoms among older persons.^[2–4]^

Recently, depression can effectively be treated through music therapy. In previous music therapy trials for depression, diverse clinical techniques relying on multiple methods have been employed.^[5]^ Music interventions for older adults are based on the notion that music elicits emotional responses and helps to retrieve memories, with recent support from research suggesting that brain regions responsible for processing music, particularly known familiar songs, may be spared even in late-stage dementia. They are offered in individual, group, and community settings.^[6]^

Tai Chi, a form of Chinese Martial Arts in existence for hundreds of years, is increasingly being used in mental health settings. Meta-analytic reviews indicate favorable outcomes on a range of psychological well-being measures including depression and anxiety, with large effect sizes for both conditions.^[7]^ In 1 randomized controlled trial conducted in an Asian elderly population, Tai Chi had a positive effect on reducing depressive symptoms compared with no treatment in older patients with depression.^[8]^ Tai Chi has also been evaluated as a complementary modality to pharmacotherapy for geriatric depression, with combination treatment showing greater reduction in depressive symptoms than pharmacotherapy alone.^[9]^

However, it is still controversial whether combined music and Tai Chi therapy will be more effective than treatment alone for patients with major depression.^[10]^ Based on the separate effectiveness of Tai Chi and music mentioned above, we combined Tai Chi with soft relaxing Chinese folk music to determine whether a synergistic effect would be present in the control of depressive symptoms among community-dwelling persons in China. Therefore, the primary objective of the present study was to evaluate the efficacy of Tai Chi and music therapy on psychological status and quality of life among patients with major depressive disorder in China.

## 2 Materials and methods

### 2.1 Ethical approval

This work is a part of a comprehensive research project to assess and provide intervention that potentially improves psychological status and quality of life among patients with major depressive disorder. This research project has been registered in the research registry (with number: researchregistry6597) and received ethical approval from the Medical Research and Ethics Committee in Taizhou Second People's Hospital (no. tzey2020023). After the introductory briefing, the acceptance to take the presession questionnaire implied the participant's consent to participate in the study.

### 2.2 Study population

Participants recruited into the study need to fulfill the following criteria: aged 18 to 70 years; residing in the targeted communities for at least 1 year; mentally adequately alert to complete the intervention as judged by trained researchers; with a GDS score of 11 to 25 (inclusive of 11 and 25), and agreeable to take part in entire intervention process. Participants with the following characteristics are excluded: individuals who are bedridden, or with severe hearing or visual impairment leading to difficulty completing the intervention; those with a stroke or cardiovascular event within the past 6 months and the presence of other conditions which require medical assessments prior to physical exercise; individuals with a GDS score of 26 or greater (severely depressed); those who are receiving medical treatment for depression; those who exercise more than 3 times per week.

### 2.3 Random allocation

**Figure 1:**
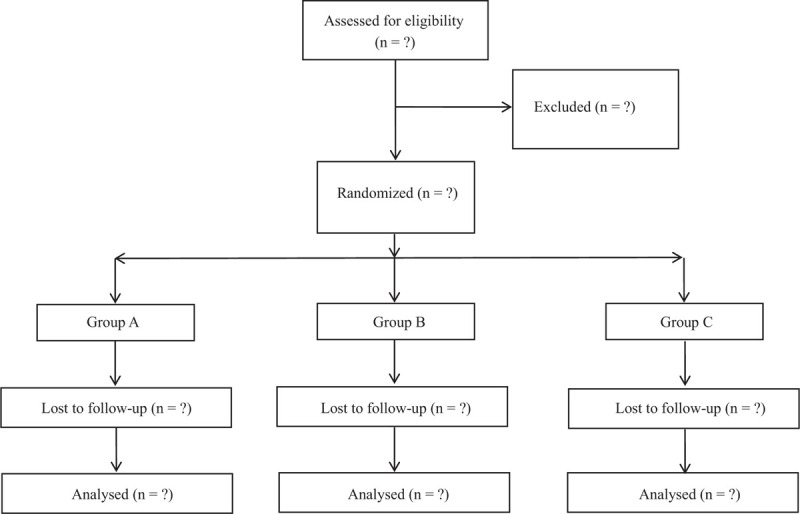
Consolidated standards of reporting trials (CONSORT) diagram of patient flow through the study.

Before starting the study, a randomization list is produced using software-generated randomized numbers; the randomization depends on random blocks of 10. Eligible participants are divided into 3 groups according to completely randomized design: combined group (music therapy + Tai Chi), music therapy alone group and Tai Chi alone group (Fig. 1). The program duration is 12 months. Participants are enrolled by the research assistant. After baseline examination, all patients will be given a full explanation of the treatment protocol and will be required to sign a written informed consent for study participation and for the publication of the results. All the data collectors, statistical analysts, as well as result assessors are not aware of grouping assignment (Fig. 1).

### 2.4 Intervention and control protocols

The music therapy group is provided by a trained music therapist, who is registered with the appropriate professional association or registration body in his or her country and should also be skilled as a musician. To facilitate individual relationship-building, the music therapist will offer each resident an initial 20 minutes assessment with the aim of determining their musical preferences and starting to build individual rapport. The music therapist will also use other sources to determine the participants’ musical biography, cultural background, history, personal strengths, resources and disabilities, and any other information that could be useful to bring into music therapy sessions.

Tai Chi is taught as a 24-form Yang style; instruction is given 5 days per week for 1 h for 12 weeks. The Tai Chi group is led by an experienced practitioner who will demonstrate a set of slow, nonstrenuous movements coordinated with deep breathing. Participants are taught traditional Sun and Yang styles of Tai Chi. Instructors will take participants through warm-up exercises for 5 minutes, Tai Chi movements and form for 20 minutes and cool down exercises for 5 minutes. At the end of the 12-week period, participants are encouraged to continue Tai Chi, either alone or via a community group; however, no assistance is provided by the investigators during this period.

### 2.5 Outcomes

Questions on the patients’ sociodemographic characteristics concerned their age, sex, marital status, level of education, and occupation type (Table 1). Quality of life is measured using the Chinese version of the WHOQOL-BREF questionnaire. The WHOQOL-BREF consists of 26 items, scored on a 5-point Likert scale of 1 to 5, with an item score of 1 indicating “not at all” or “very poor/dissatisfied” and an item score of 5 indicating “very good,” “completely,” or “extremely.” There are 2 general items regarding health conditions which would be analyzed independently. The other items evaluate quality of life (QOL) in 4 main domains: physical health (7 items), psychological (6 items), social relationships (3 items), and environment (8 items). Three items are phased negatively and hence, scored in reverse. The domain scores are computed separately and the raw score of each domain is converted into a 0 to 100 transformed score, according to the WHOQOL user manual. After conversion, the maximal scores for the physical health, psychological, social relationships, and environment domains are 100 respectively, with a higher score indicating a better QOL. Satisfactory reliability and validity of WHOQOL-BREF have been demonstrated in the Chinese population with a Cronbach-a coefficient of 0.892.

**Table 1:** Patient baseline demographics.

Demographics	Combined group	Music group	Tai Chi group	*P* value
Number of patients				
Age^∗^ (y)				
Male sex (no. [%])				
BMI^∗^ (kg/m^2^)				
Follow-up^∗^ (mo)				

BMI = body mass index.

∗ The values are given as the mean and the SD.

### 2.6 Statistical analysis

The analyses will be performed using SPSS 22.0.0 (SPSS Inc, Chicago, IL). Frequencies and descriptive statistics will be used for patients’ demographic presentation, while means and standard deviations will be calculated for the continuous variables and group differences will be analyzed by using Pearson *χ*^2^test for categorical variables. Kolmogorov–Smirnov test will be applied to check the distribution of data. Independent *t* test will be used in case of normally distributed data, whereas Mann–Whitney *U* test for non-normal distributions. *P* < .05 will be considered significant for all analyses.

## 3 Discussion

Depression is a common health problem and an increasing global burden. WHO has found unipolar depression to be the third largest burden of disease globally in 2004 and is projected to rank first in 2030. A result of depression is the loss of social and cognitive functions and quality of life. One of the symptoms in depression is reduction in sleep quality (insomnia). Sleep disturbances associated with depression include difficulties in falling asleep and maintaining sleep.^[11]^ Cognitive behavioral therapy is recommended in clinical guidelines as first-line treatment. Other treatment modalities for sleep promotion include sleep hygiene, physical activity, light therapy, relaxation techniques, music/nature sounds, and acupuncture. Resolving sleep disturbances in patients with active or previous depression is important as it may prevent worsening of symptoms and relapse of depression.^[12]^

However, it is still controversial whether combined music and Tai Chi therapy will be more effective than treatment alone for patients with major depression. Based on the separate effectiveness of Tai Chi and music mentioned above, we combined Tai Chi with soft relaxing Chinese folk music, to determine whether a synergistic effect would be present in the control of depressive symptoms among community-dwelling persons in China. Therefore, the primary objective of the present study was to evaluate the efficacy of Tai Chi and music therapy on psychological status and quality of life among patients with major depressive disorder in China.

## Author contributions

Huixian Xie and Yiping Tang conceived, designed, and planed the study. Huixian Xie and Yiping Tang are recruiting the study participants and performing the interventions. Fengmin Cheng supervised the study. Huixian Xie and Yiping Tang will interpret and analyze the data. Huixian Xie and Yiping Tang drafted the manuscript. Fengmin Cheng and Jucai Chu critically revised the manuscript for important intellectual content. All authors have full access to the manuscript and take responsibility for the study design. All authors have approved the manuscript and agree with submission.

**Conceptualization:** Yiping Tang.

**Data curation:** Huixian Xie, Yiping Tang.

**Formal analysis:** Huixian Xie, Yiping Tang, Fengmin Cheng.

**Funding acquisition:** Jucai Chu.

**Investigation:** Huixian Xie, Yiping Tang, Fengmin Cheng.

**Methodology:** Huixian Xie.

**Project administration:** Jucai Chu.

**Resources:** Jucai Chu.

**Software:** Yiping Tang, Fengmin Cheng.

**Supervision:** Jucai Chu.

**Validation:** Yiping Tang.

**Visualization:** Huixian Xie.

**Writing – original draft:** Huixian Xie.

**Writing – review & editing:** Fengmin Cheng, Jucai Chu.
